# Peptide-Based Electrospun Fibers: Current Status and Emerging Developments

**DOI:** 10.3390/nano11051262

**Published:** 2021-05-11

**Authors:** Raffaella Bucci, Evangelos Georgilis, Alexander M. Bittner, Maria L. Gelmi, Francesca Clerici

**Affiliations:** 1 Department of Pharmaceutical Sciences, University of Milan, Via Venezian 21, 20133 Milan, Italy; marialuisa.gelmi@unimi.it (M.L.G.); francesca.clerici@unimi.it (F.C.); 2 CIC nanoGUNE, (BRTA) Tolosa Hiribidea 76, 20018 Donostia-San Sebastián, Spain; e.georgilis@nanogune.eu (E.G.); a.bittner@nanogune.eu (A.M.B.); 3 Ikerbasque Basque Foundation for Science, Pl. Euskadi 5, 48009 Bilbao, Spain

**Keywords:** peptides, peptidomimetics, peptide-based electrospun nanofibers, self-assembly, electrospinning

## Abstract

Electrospinning is a well-known, straightforward, and versatile technique, widely used for the preparation of fibers by electrifying a polymer solution. However, a high molecular weight is not essential for obtaining uniform electrospun fibers; in fact, the primary criterion to succeed is the presence of sufficient intermolecular interactions, which function similar to chain entanglements. Some small molecules able to self-assemble have been electrospun from solution into fibers and, among them, peptides containing both natural and non-natural amino acids are of particular relevance. Nowadays, the use of peptides for this purpose is at an early stage, but it is gaining more and more interest, and we are now witnessing the transition from basic research towards applications. Considering the novelty in the relevant processing, the aim of this review is to analyze the state of the art from the early 2000s on. Moreover, advantages and drawbacks in using peptides as the main or sole component for generating electrospun nanofibers will be discussed. Characterization techniques that are specifically targeted to the produced peptide fibers are presented.

## 1. Introduction

The development of biomaterials to improve human life is one of the hottest topics in materials science. The use of peptides for their preparation is gaining more attention due to the biocompatibility, the possibility to tune their physical features by modulating the amino acid sequence, and for the applications, ranging from tissue and biosurface engineering to drug delivery systems, from components for biosensing to bioanalytical devices [1,2].

Recently, there has been considerable interest in nanofiber films and nanofiber mats, produced by the electrospinning technique, a well-known process to tune (nano)fiber diameters (from nanometers to micrometers) [3]. This technique is traditionally used to form fibers from high molecular weight synthetic polymers, but it has recently been extended to supramolecular assemblies, such as surfactants, host–guest complexes, cyclodextrins, and peptides [4]. For the sake of simplicity, we will classify amino acid sequences shorter than 50 amino acids as peptides or peptidomimetics, in case an unnatural amino acid or a scaffold is present in the peptide sequence. Polypeptides refer to larger sequences. Our analysis is based on fibers formed by amino acids with both ultrashort and long sequences (i.e., polypeptides). Electrospinning of proteins is not considered because it has been extensively reviewed, and advantages and disadvantages are discussed in considerable depth elsewhere [5,6,7,8,9].

The promising platforms mentioned above are now driving various applications. Among them, tissue engineering is probably the most explored area [10,11]. In this case, biodegradable scaffolds are needed to direct tissue repair and regeneration, while providing temporary structural support for cells. Natural and engineered proteins (collagen, silk fibroin, elastin, elastin-like polypeptides) [12,13,14] are used to reach this target [7,15], as well as pure polymers [7] and polymer/peptide blends or constructs. The constructs are based on bulk or surface modification, electrostatic attraction [16,17,18], or covalent linking, prior or after the electrospinning process [19].

On the other hand, a good compromise between natural and synthetic polymers for biomedicine and biotechnology applications is represented by the use of synthetic or recombinant peptides, which have several advantages, including reducing or eliminating animal-source materials [19,20].

The use of poly- [21] and oligo-peptides [22,23,24,25,26] in electrospinning has been reported, focusing on applications in superhydrophobic surface development and rigid scaffolds for tissue engineering. Very recently, it was demonstrated that short peptides containing both natural or non-natural amino acids can be electrospun into solid, continuous, homogenous, and bead-free quasi-endless micro- and nanofibers [27]. However, generally, the production of electrospun pure peptide fibers is at a rather early stage.

Moving the electrospinning technique from proteins or synthetic polymers to pure (poly-)peptides holds tremendous promises in life sciences, but it can also produce materials which can be utilized beyond biological applications, directed for important purposes in fields as diverse as energy [28] and environmental aspects [29].

**Figure 1:**
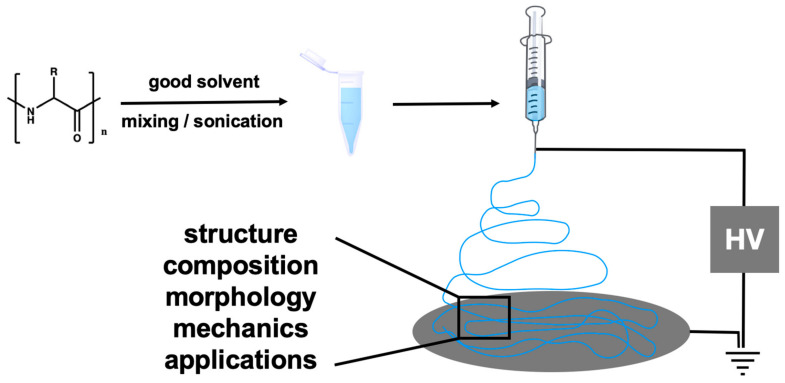
Schematic representation of peptide fiber formation via electrospinning.

Considering the novelty of this approach, the aim of this review is to analyze (from the early 2000s onwards) the state of the art in using peptides as the main or sole component for generating electrospun micro- and nanofibers. A schematic representation the of electrospinning technique is depicted in Figure 1.

## 2. Peptides in Electrospinning

There are several benefits in using peptides for electrospinning [26] which we should emphasize. Firstly, peptides can be specifically selected or designed to meet the criteria of biocompatibility required for biomedical applications (ranging from regenerative medicine, such as scaffolds for blood vessels, bone tissue engineering, and drug delivery) [25,30]. Moreover, common polymer synthetic strategies yield a distribution of molecular weights which may differ for every production [31]. Such batch-to-batch differences are undesirable, especially for the development of nanomedical platforms. In typical polymer blends, the presence of more than one compound can be an issue for the reproducibility of electrospinning [32]; moreover, the compounds can segregate into domains. On the other hand, large-scale peptide synthesis can currently reach up to kilogram yields, which is lower than polymer synthesis. However, the synthetic methods are constantly being optimized for the sustainable production of peptide compounds with high purity, at reduced cost [33,34,35]. The greatest disadvantage is that peptide electrospinning requires a highly viscous solution (usually 1 to 10 Pa·s), which can only be achieved by high concentrations of 10–50 wt.%, while most polymers merely require 2–10 wt.%. Together with the required high vapor pressure, the selection of solvents is very restricted. Therefore, chaotropic fluorinated solvents (e.g., 1,1,1,3,3,3-hexafluoro-2-propanol (HFIP) and trifluoroacetic acid (TFA)) have been extensively employed to dissolve peptides prior to electrospinning. However, benign solvents such as acetic acid and aqueous solutions are being investigated as green, user-friendly alternatives [36,37,38,39,40].

Electrospun nanofibers based on non-cytotoxic and bioactive peptides are the ideal candidates for biological applications, because they are biodegradable, capable of promoting cell–substrate interactions, and chemically compatible with aqueous solutions and physiological conditions [28,29]. Formed by at least 20 coded amino acids, peptides possess a high chemical diversity tunable for different biological targets [22,41]. The main drawback is their lack of proteolytic stability. This issue can be overcome by using peptidomimetics, compounds containing non-natural amino acids or non-peptidic structural elements, capable of mimicking the actions of a natural parent peptide [42,43].

Focusing on short peptides and peptide-based compounds, we should emphasize that advances in peptide electrospun nanofibers have brought significant information on the forces involved in peptide assembly. Compared to “natural self-assembly”, electrospinning is based on a “forced” assembly, because the trigger for the formation of the material is the application of an electrical field [25,41]. It was found that those peptides which naturally self-assemble into fibers or tubes are also prone to forming electrospun fibers. This intrinsic property is due to a high anisotropy, a key feature of peptides. Indeed, they could be characterized by different secondary structures depending on their amino acid sequences [41]. The secondary structures determine the 3D folded structures, formed by non-covalent interactions that are at the base of the aggregate formation [43,44,45]. In conclusion, very often, peptides with a stable conformation are able to self-assemble, and this is also one of the most important factors for the formation of well-defined electrospun fibers [41].

### 2.1. Short Peptides

#### 2.1.1. Peptides Containing an Aromatic Moiety

Currently, only few reports of the electrospinning of short peptide sequences have been published. The pioneers were probably Singh et al. in 2008, whose contribution in this field became of relevance thereafter [24].

**Figure 2:**
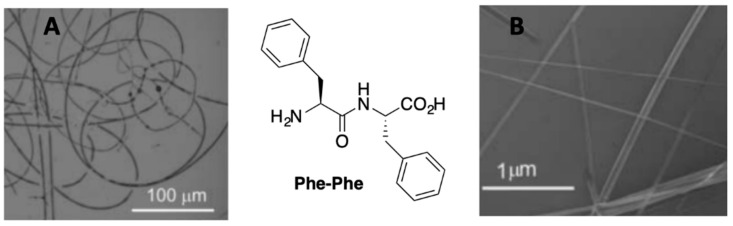
Phe-Phe electrospun nanofibers of (**A**) Phe-Phe self-assembled nanotubes of (**B**). Reproduced from [24] with permission from John Wiley and Sons.

In particular, they first focused on short peptides containing aromatic amino acids, it being known that π–π interactions play a vital role in stabilizing the structures of the bulk crystal and of fibers [46,47]. Diphenylalanine (Phe-Phe) is very well studied in materials science due to its capability to self-assemble into various architectures. It is the central core of amyloid polypeptides, being the cause for fibril formation in Alzheimer’s disease [48]. After different attempts, dissolution of the dipeptide hexafluoroisopropanol (HFIP, boiling point 59 °C) at 17.5 wt.% and the application of 13.2 kV was found to be optimal, allowing to obtain fibers of “infinite” length (Figure 2). Due to the promising results, a thorough characterization of the obtained fibers was performed in comparison to self-assembled Phe-Phe nanotubes as a control material [48]. Interestingly, Raman spectra of both materials showed the same resonances. In order to demonstrate that the surface of the electrospun fibers was composed by only phenyl moieties, the authors studied the wetting behavior of the new material. When some droplets of paraffin were placed on the fibers, a thin oil film moved along the fibers, while when the same amount of water was used, no flow was observed. This concept is in agreement with the Görbitz structural model of Phe-Phe, explaining that the electrospun nanofibers are formed based on π–π interactions of the aryl moieties, in the same way as self-assembled Phe-Phe nanotubes [46,49].

**Figure 3:**
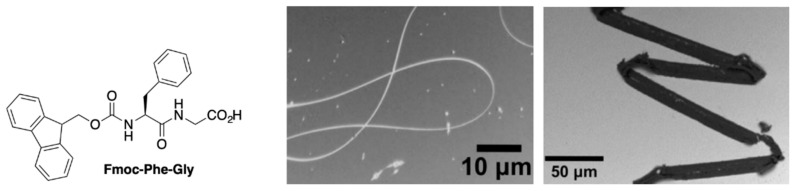
Band-like morphology of Fmoc-Phe-Gly electrospun nanofibers. Reproduced from [41] with permission from The Royal Society of Chemistry.

The same authors succeeded in the formation of long electrospun fibers, without any significant defects, using solutions of the *N*-terminus-protected peptides Fmoc-Gly and Fmoc-Phe-Gly [24]. To characterize them, IR and Raman experiments were performed, and the spectra were compared with the theoretical result obtained by Quantum Chemical Computational Studies. Raman spectroscopy was used to elucidate the non-covalent interactions at the base of the formation of peptide-based electrospun fibers. It was proven that the formation of hydrogen bonds, together with π–π stacking, are driving forces of the fiber construction. Moreover, the authors claim the importance of Raman spectroscopy for the straightforward analysis of the fibers, allowing for simple detection of H-bonding and (less clearly) of π–π interactions [24]. Electron microscopy here shows a band-like morphology with a 10 μm diameter for Fmoc-Phe-Gly electrospun fibers (Figure 3) [41].

More recently, Hamedani et al. described the capability of tyrosine-based dipeptides to yield electrospun nanofibers of good quality (i.e., bead-free fibers without the simultaneous presence of droplets) [25]. The aim of this work was the preparation of new biodegradable and non-toxic fibers as potential biomedical candidates for tissue repair, drug delivery, or coating for implants. The authors focused on tyrosine-based peptides because the presence of tyrosine and dityrosine crosslinks in proteins, such as resilin in cuticles of many insects, contributes to their mechanical stability due to the high elastic modulus [50,51].

**Figure 4:**
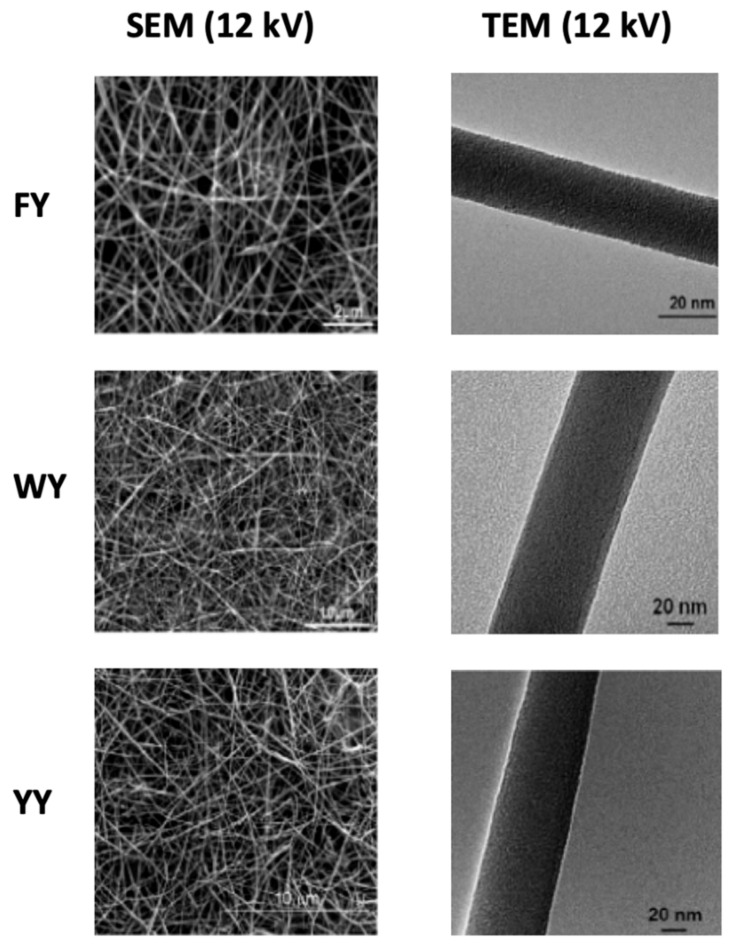
Scanning and transmission electron micrographs SEM and TEM micrographs of Phe-Tyr (FY), Trp-Tyr (WY) and Tyr-Tyr (YY). Reproduced from [25] with permission from The Royal Society of Chemistry.

Three dipeptides, i.e., Phe-Tyr, Trp-Tyr and Tyr-Tyr were selected, and different attempts were performed to find the optimal conditions for the electrospinning. The optimized conditions (Table 1) yielded fibers without macroscopic imperfections (Figure 4).

The three compounds behaved differently under the influence of the voltage: the decrease in the voltage reduces the diameters of the Phe-Tyr and Trp-Tyr nanofibers, thereby also improving their morphology (the beads in the nanofibers disappear). This effect was observed in prior studies involving polymers such as poly(vinyl alcohol) and poly(ethylene oxide) [52]. On the contrary, the Tyr-Tyr nanofiber diameter decreased with increasing voltage range during the deposition process. In this case, ultra-thin fibers were formed. For the three dipeptides, transmission electron microscopy demonstrated the smoothness and cylindrical morphology of the fibers created at 16 kV (Figure 3), making them suitable for biological applications [25].

Tyrosine-based materials are expected to have a high elastic modulus (see Section 3.7); therefore, this parameter was analyzed by nanoindentation. The peptide nanofibrous mats exhibit stiffnesses in the range of 0.5–4 GPa. Even though the Tyr-Tyr nanofibers were found to have the lowest stiffness (0.5 GPa) in all the tested electrospun nanofibers, the stiffness is high compared to other electrospun biodegradable polymeric fibers [53]. Finally, IR and Raman spectroscopy analyses confirmed the importance of π–π interactions and hydrogen bonds for the formation of electrospun nanofibers.

#### 2.1.2. Amphiphilic Peptides

Amphiphilic molecules such as phospholipids are composed of a polar head group and an aliphatic tail, and they spontaneously form micelles and bilayers. McKee et al. were the first to develop electrospun fibers from phospholipids in the early 2000s [54]. Moving to amphiphilic peptides, the polar head group is replaced by an amino acid sequence, thus generating a class of self-assembling biomolecules [55,56] extensively investigated in materials science for a range of biomedical applications, from spinal cord injury repair [57] to wound healing [58].

**Figure 5:**
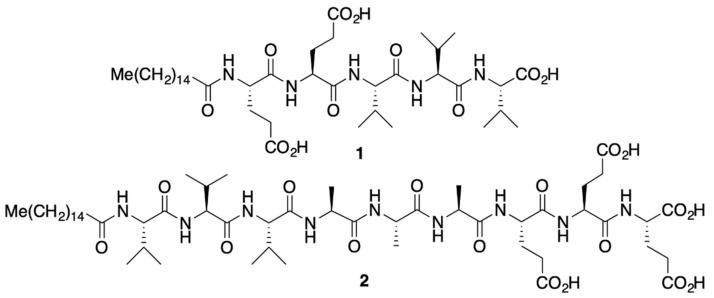
Chemical structures of compounds **1** and **2**.

Tayi et al. reported the electrospinning of mid-sized amphiphilic peptides (from five to nine AAs) for the formation of bioactive surfaces for implantable devices, sutures, and scaffolds for tissue regeneration [59]. In particular, they chose two amphiphilic peptides (Figure 5).

Before the electrospinning, the self-assembly of peptides **1** and **2** (Figure 5) in water was investigated. Electron and optical microscopy were used to observe the morphology of the assemblies. In particular, compound **1** formed a twisted, ribbon-like nanostructure with a width of 30 nm and a periodicity of 300 nm, while **2** formed long nanofibers with lengths exceeding 10 μm and widths of approximately 7 nm. Several studies on different solutions of peptides **1** and **2** were carried out before focusing on the electrospinning.

To determine the appropriate conditions for electrospinning, the viscosity, surface tension, and solution conductivity were measured.

It is known that high viscosities are required for electrospinning. Rheological studies of **1** and **2** were performed from low concentrations (0.2 wt.%) up to 3 wt.% in Milli-Q water, using, as a control compound, poly(ethylene glycol) (PEG), a polymer commonly adopted for electrospinning experiments [60]. The optimal viscosity for compounds **1** and **2** was found at 3 wt.% (~2 Pa·s at low shear rates).

While surfactants are sometimes added to the electrospinning solution in order to lower the surface tension, amphiphilic peptides behave as surfactants themselves. The surface tension of compound **1** was 45 mN/m at 3 wt.% and it was 58 mN/m at 3 wt.% for compound **2**, comparable to values for PEG solutions (for pure water it is 73.8 mN/m).

As highlighted before, solution conductivity is the other parameter to take into account for a good morphology of electrospun nanofibers [61]. Unmodified peptides, with their carboxylate and protonated amine groups, are usually zwitterionic in solids and often also in solution. Depending on the pH of the solution and the pKa of the residues, peptides are charged when dissolved, and the charge affects droplet formation during the electrospinning and the solution conductivity. Due to the presence of glutamic acid residues and C-terminal carboxylic acid, the solution of compounds **1** and **2** showed conductivities of 2.6 and 3.6 mS/cm at 3 wt.%, respectively, far exceeding that of the PEG-based control [59].

**Figure 6:**
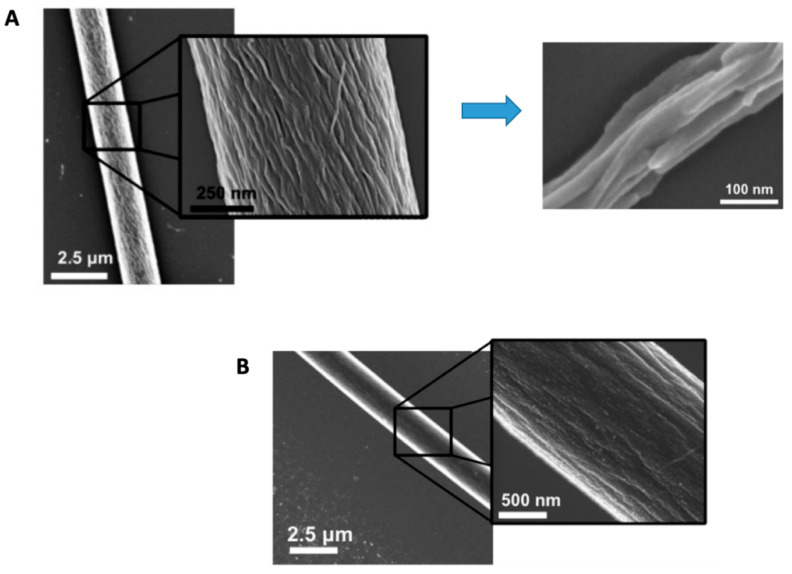
SEM electron micrographs of electrospun nanofibers of compound **1** (**A**) and compound **2** (**B**). Reproduced from [59] with permission from the ACS (https://pubs.acs.org/doi/10.1021/bm401877s (accessed on 10 May 2021)). Further permissions related to the material excerpted should be addressed to the ACS.

Taking into consideration the ability of these two peptides to self-assemble, the authors claimed the concrete possibility of obtaining good electrospun nanofibers with an appropriate concentration of 3 wt.% [58]. A voltage of 10 kV with a flow rate of 0.04 mL/h was used. Electrospun microfibers were obtained for both peptides without the addition of polymers or surfactants (Figure 6). The fibers had similar diameters, 3.8 ± 0.4 and 3.9 ± 1.3 μm, respectively, but different morphologies: compound **1** formed nanofibers with aligned nanoribbon-like structures, while compound **2** produced fibers composed of bundles of cylindrical nanofibers of approximately 50 nm in width (Figure 6).

Similar to conventional polymeric materials, these two types of electrospun nanofibers can be deposited on a large variety of collectors/substrate surfaces, from medical devices to glass or silicon. As applications, electrospun peptide amphiphile fibers can be used to improve cell adhesion to surfaces or to elicit a tailored biological response to medical devices by utilizing bioactive epitopes. Thus far, electrospun polymer fibers have been coated with peptide amphiphiles bearing an RGD cell adhesion motif in order to create biomimetic scaffolds for tissue engineering applications [62,63]. It is therefore expected that the electrospinning of peptide amphiphiles will be further employed to obtain fibers without the use of polymer carriers.

### 2.2. Peptidomimetics

**Figure 7:**
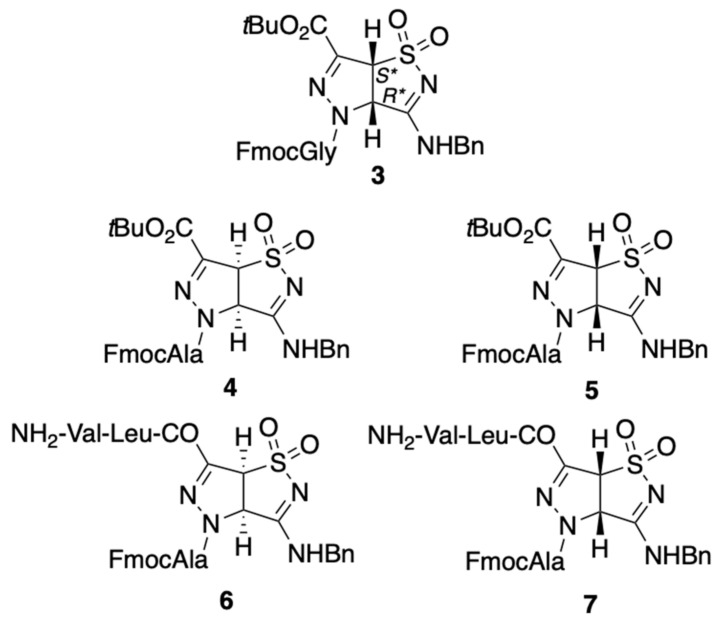
Chemical formulae of peptidomimetics **3**–**7**.

As emphasized before, the main drawback in using natural peptides is the lack of stability under a proteolytic environment. To avoid this problem, a non-natural amino acid or a synthetic scaffold can be inserted in the peptide backbone, forming a peptidomimetic, a molecule that possesses the features of the target peptide and stability against proteases [64,65,66,67,68]. Recently, Locarno et al. reported the synthesis of a β-amino acid-like scaffold, characterized by a pyrazole–isothiazole core, which was inserted in different short peptide sequences. The aim was to evaluate a possible effect of the different decorations and different stereochemistries of the molecules [27] (Figure 7). The spinnability of five pyrazole–isothiazole derivatives, compounds **3**–**7**, was investigated.

**Figure 8:**
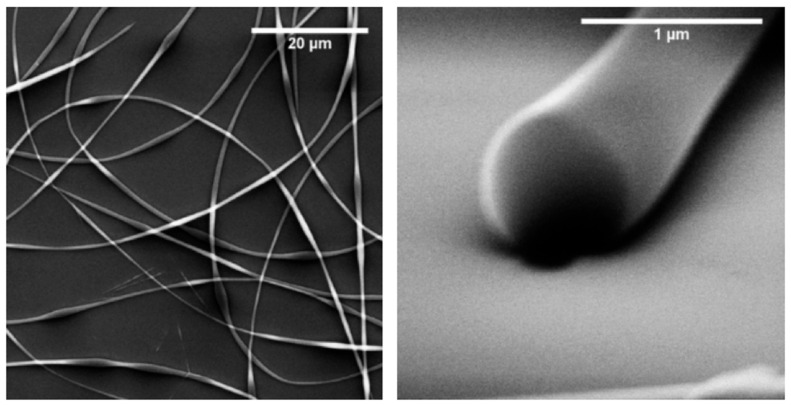
SEM images of electrospun fibers of compound **3**. Reproduced from [27] with permission from The Royal Society of Chemistry.

Electrospinning of compound **3** in HFIP (30 wt.%.) gave continuous fibers (600 nm diameter), which were fairly homogenous and without any beads. SEM confirmed the formation of fully filled fibers (Figure 8).

The other compounds did not present the same spinnability: compound **4** showed homogeneous fibers with defects; the corresponding diastereoisomer **5** was not soluble at all in HFIP; compound **6** could not be spun; and compound **7** gave only short and discontinuous nanofibers.

IR and Raman experiments proved that intermolecular hydrogen bonds, but mostly π–π stacking, are at the basis of the formation of the electrospun nanofibers of compound **3**. Moreover, this new material could be an interesting candidate for biomedical applications, because the electrospun nanofibers are not cytotoxic up to a concentration of 200 μM [27].

### 2.3. Polypeptides

The importance of polypeptides (see Table 2 for acronyms) in the electrospinning field needs to be highlighted because many examples of electrospun materials based on them is reported. In this context, it should be noted that polypeptides do rarely occur in nature, but that peptide repeats are very common. Translated to polymer science, one could name them “copolymers”, but with a rather large number of different “monomers”.

Few examples regarding the production of electrospun fibers of poly(-alanine) [69,70] or poly(-aspartic acid) [71] are reported. On the other hand, PGA- and PBG-based nanofibers have extensively been used for tissue engineering, as homo- or as copolymers, for various applications, from biomedicine to novel piezoelectric materials [72] and wettable solid supports [73].

For this purpose, synthetic model polypeptides, i.e., polycationic PLO and PLEY, were electrospun and the obtained mats were studied by circular dichroism (CD) and infrared (IR) spectroscopy, and the fiber composition was analyzed with X-ray spectroscopy (EDX) [74].

Moreover, electrospun nanofibers of PLEY and PLO polypeptides were prepared, forming water-insoluble materials. In particular, two methods were used for in situ polypeptide crosslinking: (i) exposure of PLO to glutaraldehyde vapor (GA, 25%) [75]; and (ii) PLEY immersion in 1-ethyl-3-(3-dimethylaminopropyl)carbodiimide (EDC, in 90% ethanol/10% water; 50 mM solution) [76]. The authors demonstrated the good spinnability of crosslinked PLO and PLEY without morphological changes after cross-linking. After thorough characterization via CD and IR, they observed that PLEY and PLO themselves did not adopt a stable secondary structure in aqueous solution, while after electrospinning, the fibers and films showed a β-sheet conformation prior to the crosslinking. On the other hand, electrospun fibers of both crosslinked polymers showed an irregular backbone structure.

These two fiber mats represent interesting wettable substrates, on which fibroblasts could grow in the presence of serum. Such behavior is relevant to fiber mat applications in biotechnology and medicine; in vitro tissue engineering, ex vivo stem cell therapy, wound healing, and biomaterial implantation, for instance [74].

**Figure 9:**
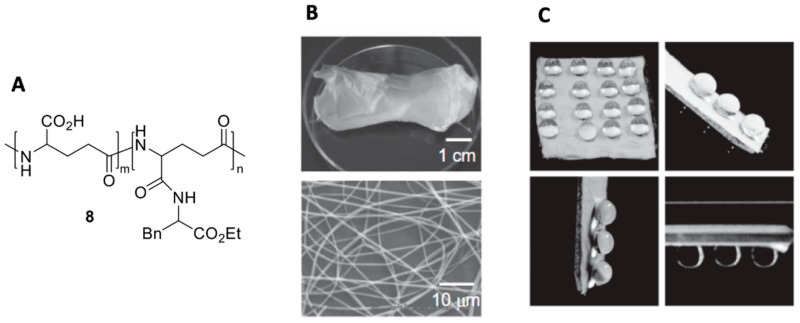
(**A**) Chemical formula of modified γ-PGA **8**. (**B**) Electrospun materials of compound **8**. (**C**) Strong adhesion of water droplets (“inversion test”). Reproduced from [21] with permission from John Wiley and Sons.

The concept of superhydrophobicity, observed on a lotus leaf or on a rose petal, has fascinated scientists in many fields, who are trying to produce functional materials with this particular feature [77,78]. Their interest derives from the potential application in green chemistry, but also in biomedicine and environmental applications [79]. Two types of superhydrophobic surfaces are known in the literature, depending on their properties: a lotus effect surface and a petal effect surface [80,81]. The first shows repulsion properties, as a “water-walking phenomenon”, while the second possesses high adhesive properties similar to water droplets on a rose petal. Yoshida et al. investigated γ-PGA electrospun nanofibers modified with hydrophobic L-Phe (compound **8**, Figure 9A) as superhydrophobic and biodegradable materials [21].

The best results in terms of electrospinning were obtained using 20 wt.% of γ-PGA-Phe-80 solution in HFIP with an applied voltage of 20 kV, collection distance of 20 cm, and a flow rate of 0.50 mL/h. The formed fibers were homogeneous with diameters of 660 ± 220 nm, and fibrous mats could be easily peeled off from the substrates after 2 h electrospinning (Figure 9B). It must be pointed out that water droplets remain stuck to the material surface even if it is inverted, clearly indicating a petal-type superhydrophobic property. The wetting property was easily tuned, depending on the number of hydrophobic moieties. The remaining functional groups can be employed for on-demand chemical modifications. This represents the first example of a biodegradable electrospun materials with petal-type superhydrophobicity.

**Scheme 1:**
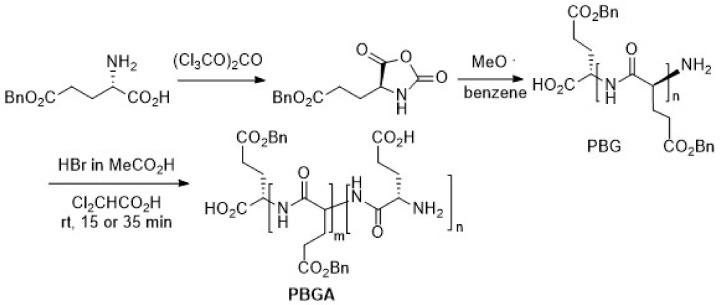
Synthetic pathway of PBG and PBGA.

In 2018, Wang et al. reported on the synthesis of a poly(γ-benzyl-l-glutamate)-r-poly(glutamic acid) random copolymer and its use in the formation of electrospun fibers, used for neural regeneration [82]. In particular, three novel polypeptides belonging to the poly(glutamate) class were used to fabricate the material: PBG, PGA, PBGA20 and PBGA30 (Table 2); their synthesis is described in Scheme 1. In the first step, L-glutamic acid-γ-benzyl ester was treated with triphosgene, affording the monomer benzyl-Glu-NCA. This latter compound was left to react with a sodium methoxide initiator in benzene. Finally, the copolymers PBGA20 and PGA30 were obtained after hydrolyzing the PBG for 15 and 35 min, respectively. The molar percentage of poly(-glutamic acid) in the PBGA20 and PBGA30 was determined by NMR.

**Figure 10:**
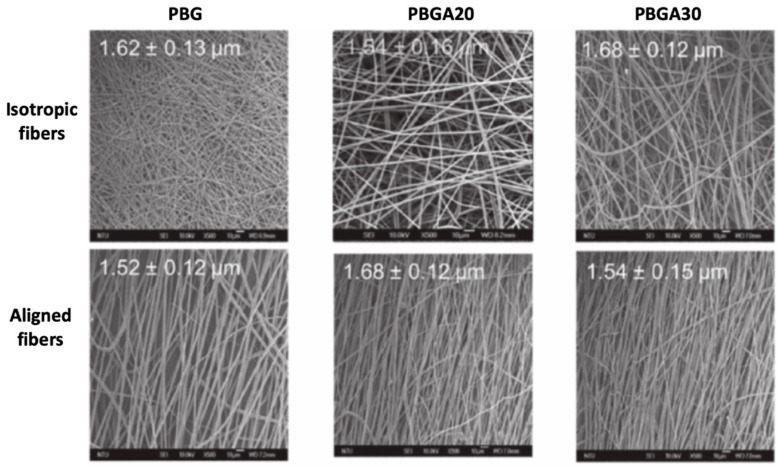
SEM photos of 3D scaffolds of poly(glutamates) fabricated from isotropic fibers (**top**) and aligned fibers (**bottom**). Reproduced from [82] with permission from John Wiley and Sons.

The optimal electrospinning conditions were found to be 20 wt.% of the polymer in a mixture of THF/DMAc (9:1), spun with a flow rate of 5 mL/h at 20 kV. Two collection modes were used to obtain two types of electrospun nanofibers (diameter within 1−2 μm): (i) isotropic fibers, collected as usual on a metal plate; and (ii) aligned oriented fibers deposited on a rotating drum (Figure 10).

Interestingly, both biocompatibility and neurite growth on the fibrous scaffold improve with increasing glutamic acid content in the polymer. Moreover, the orientation of the fibers is also important: aligned fibers can guide anisotropic growth of neurites along the fiber into 3D domains better than randomly oriented fibers [83].

The same research group found a more promising polypeptide for neural tissue engineering and drug-screening platforms [84]. PBGA20 was transformed into the corresponding salt PBGA20-Na that promotes longer neurite outgrowth compared with the PBG and PBGA20. Furthermore, electrical stimulation of the cells on the PBGA20 and PBGA20-Na scaffolds induced an enhancement of adhesion, proliferation, and differentiation.

PBG polypeptide alone is also known as a matrix of piezoelectric materials. Piezoelectricity is the electric charge that accumulates in certain solid materials (such as crystals, certain ceramics, and biological matter such as bone, DNA, and various proteins) in response to applied mechanical stress, making piezoelectric materials interesting for the creation of flexible structures [83].

Pan et al. investigated the piezoelectricity of PBG polypeptide using near-field electrospinning (NFES) [85]. This technique employs very short distances from the electrified orifice/needle tip to the collection surface. NFES enables the production of continuous fibers of sub-micrometer diameter, deposited in preselected lines on a flat surface [86]. It has been developed to achieve better results than the fiber deposition geometry. PBG polypeptide was chosen because it has a stable α-helix secondary structure in solution. The hydrogen bonds that stabilize the conformation are aligned parallel to the central axis and under a permanent dipole force; thus, the amino acidic residues can form dipoles arranged in high-density, transforming the fibers into piezoelectric materials.

It was demonstrated that the fibers exhibit permanent piezoelectricity and that an increase in the electric field leads to significant enhancement in the piezoelectric features as a result of the improved orientation of the α-helical structures. The authors also applied their PBG fibers to insect wings to generate voltage signals, proving that such systems have the potential for applications as sensing devices and energy harvesters [85].

Nguyen et al. described how the PBG piezoelectric property could be modulated, simply by maximizing the alignment of the electrospun fibers [87]. In particular, bundles of PBG fibers were fabricated with various levels of alignment. Samples with a constant width of 4 mm and thickness of 50 μm were used for the characterization. It was found that at concentrations of over 90%, the PBG fibers were oriented along the longitudinal fiber axes.

### 2.4. Polypeptoids

**Figure 11:**
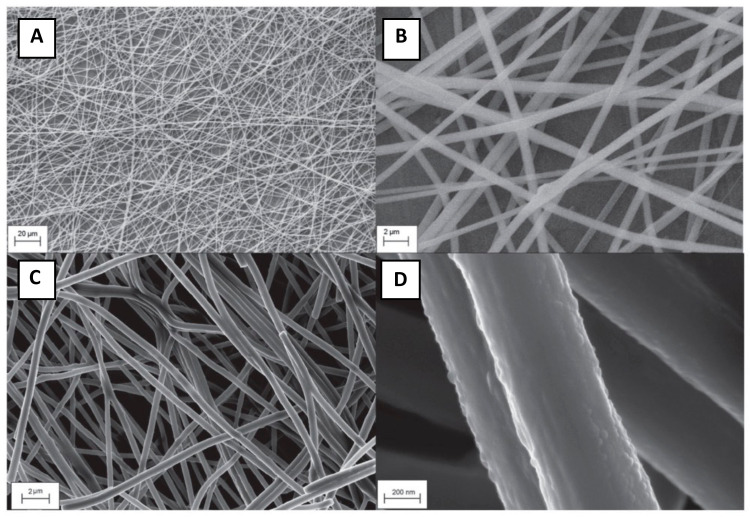
SEM images of electrospun PEG/PPGly fibers (**A**,**B**) and PPGly fibers after annealing and washing of PEG (**C**,**D**). Reproduced from [90] with permission from Wiley-VCH.

Polypeptoids (*N*-substituted poly(glycines)) represent an interesting variant of polypeptides for applications in nanomedicine and nanotechnology [88,89]. They bear a side chain on the amide nitrogen, and therefore are more resistant to proteases while retaining functionalities of a bioactive peptide. To date, the use of peptoids in electrospinning remains limited. Thielke et al. fabricated electrospun fibers of poly(*N*-(*n*-propyl) glycine) (PPGly) from a methanol/water solution containing PEG [90]. Comparing to the other systems mentioned in this work (Table 3), PPGly was the only system requiring the contribution of a second macromolecular carrier for effective spinnability. By annealing at 100 °C, PEG was washed out and pure PPGly fibers could be obtained (Figure 11), as confirmed by SEM and calorimetry. Further work on peptoids is expected to lead to the development of proteolysis-resistant, biomimetic electrospun fibers.

## 3. Characterization

**Figure 12:**
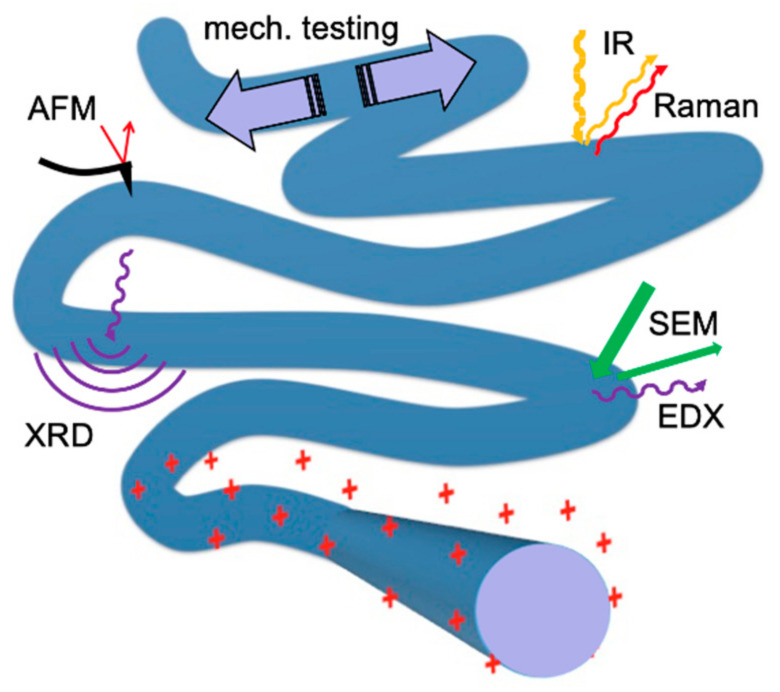
Micro- and nanoscale characterization methods. SEM, scanning electron microscopy; EDX, electron-dispersive X-ray spectroscopy; IR, infrared spectroscopy; Raman and fluorescence spectroscopy; XRD, X-ray diffraction; AFM, atomic force microscopy.

This section focuses on the characterization of a small sample of a peptide fiber mat, which is ready for application (Figure 12). Obviously, it is essential to also characterize the educt of the process, i.e., the peptide solutions by using conventional analyses (IR, NMR, CD). This is different for the produced mat: typical techniques for solid state samples are required, but the complex 3D morphology and the highly porous nature, the mechanical sensitivity, and sometimes the hydration must be taken into account.

### 3.1. Optical Microscopy

The first technique is always light (“optical”) microscopy because it provides a rapid and large-scale overview in minutes. Even when parts of the fiber are thinner than the diffraction limit, the morphology can be visualized, although its diameter cannot be measured. Confocal techniques, or simply focus adjustment, can even provide information on the 3D arrangement of the fibers or of the open space (the pores) [91]. However, the sample is often deposited on a nontransparent support. Reflection microscopy on solid surfaces can be a challenge. Indeed, the surface should be as flat as possible; typical application surfaces suffer from microscale protrusions or holes, or from striated features, all of which heavily interfere with imaging. The refractive index of the material, together with the variable fiber–surface distance, can result in huge contrast artefacts, based on coupling between light absorption and interference. Variable humidity conditions are quite easily achieved by working in a chamber, or on a cooled sample holder.

### 3.2. Scanning Electron Microscopy

Micro- and nanofibers are created; therefore, higher resolution images are practically always a must. Electron transmission techniques are optimal to measure diameters, and to detect occlusions or core–shell structures. The contrast, however, is very small if no heavy elements are present, and extremely thin fibers (<100 nm diameter) are required. Hence, scanning electron microscopy (SEM) is the method of choice [24,26,27,41,59,92,93,94]. It is generally applicable to almost all fiber morphologies and offers a nanoscale view of the outer fiber surface (see Figure 6, Figure 8 and Figure 11). It is important to keep in mind that usual SEM electrons with keV energies penetrate the fiber mat by up to tens of micrometers. SEM should thus always be considered as a rather destructive technique. The emitted electrons originate from all parts of the fiber; only when low-energy secondary electrons are detected, SEM becomes sensitive to the fiber surface, by probing only the first few nanometers. The surface must be made conductive, e.g., by sputtering ultrathin carbon or metal layers, or low voltages and currents must be used (limit around kV and nA, respectively). In some cases, the heterogeneity of the surface can be revealed by contrast effects, but more often only topographic contrast is present.

Due to the high resolution and the fast scan speed (images take merely seconds), SEM is the method of choice for a morphological analysis, even though sample exchanges are somewhat slow due to the required evacuation. It is worth noting that SEM cannot give direct information on the complete shape—the “height” (specular dimension) is only accessible upon tilting the sample (see, e.g., Figure 8) [27].

The required vacuum conditions (<1 × 10^−4^ mbar) will instantly dry any wet fiber, whether it is a peptide, or a peptide coated by a “staining” solution or by a metal layer. Another effect is charging, resulting in unstable contrast, but also in deformation. It is problematic for nonconductive samples, but also occurs on all small (sub-micrometer) asperities. Here, it is important to detect potential morphological changes. A good alternative is environmental SEM (ESEM), where the sample is not evacuated, but kept in an atmosphere of low-pressure water vapor (0.5 to 40 mbar). Sample cooling keeps fiber surfaces completely hydrated, and even wetting by water films or droplets (supersaturation) is an option [95,96], albeit not at ambient pressure. To the best of our knowledge, this technique has not yet been employed for pure peptide fibers.

Although electron-dispersive X-ray spectroscopic (EDX) analysis built into many SEMs can give useful information, this method is not straightforward, especially for low atomic numbers (C, N, and O). Beam damage (for example, deposition of carbon) can lead to huge errors.

### 3.3. Absorption Spectroscopy Methods

**Figure 13:**
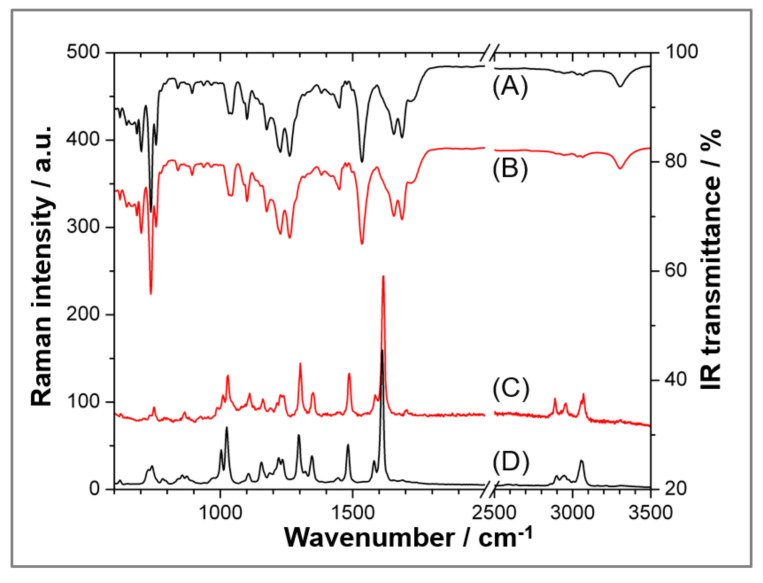
Vibrational spectroscopic analysis of Fmoc-Phe-Gly powder (black traces) and the corresponding electrospun fiber (red traces). IR spectrum of (A) powder, (B) fiber, Raman spectrum of (C) fiber and (D) powder. IR was measured in ATR configuration on a diamond prism. Raman scattering was excited at 532 nm. Reproduced from [23] with permission by Wiley-VCH.

The usual analysis method is infrared (IR) absorption spectroscopy (almost exclusively with a Michelson interferometer setup with a moving mirror for Fourier-transform IR, hence “FTIR”) [97]. It is usually applied in the mid-IR range from 25,000–2500 nm (400–4000 cm^−1^). Here, it is possible to obtain a good overview of functional groups in single compounds and in mixtures, which is impossible to achieve with simple elemental analysis [24,25,42,94,98]. IR spectroscopy always requires choosing a suitable background sample, which is normally air or the pure solid substrate on which the fiber is deposited. This makes the method rather slow (some minutes per spectrum). Generally, any solid, including the fiber itself, can cause interference effects in IR transmission for thicknesses in the micrometer range. Such effects can completely dominate and render an analysis impossible. Therefore, IR techniques based on flat substrates are often preferable; they usually require polarization of the incoming IR beam. For example, thin gold or aluminum layers are sufficient to provide almost-perfect IR reflection (differently from silicon). The experimental setup is often very simple for ATR [93]. Here, air on the ATR crystal is the background, and the sample of a fiber mat simply requires deposition on the crystal. However, ATR probes exclusively several micrometers in front the crystal, with exponentially decaying sensitivity. In order to place sufficient fiber material close to the ATR crystal surface, the mat has to be pressed against it, which might be detrimental for very brittle fibers. Figure 13 shows an example of a typical functional group analysis.

Moving to smaller wavelengths, near-infrared (NIR) spectroscopy in the wavelength range of 800–2000 nm (12,500–5000 cm^−1^) is playing an ever-larger role in pharmacology [97,98]. Here, vibrational overtones give rise to very complex spectra, which are mainly employed for fingerprinting, i.e., the correlated presence of a plethora of absorption signals is interpreted as the presence of a compound. This technique is little used in basic research, and is usually not customized for samples in the shape of mats.

It is worth mentioning UV/Vis absorption spectroscopy as a standard technique for peptide concentration determination by applying the Beer–Lambert law. The method is semi-automatic for liquids, but not straightforward for solids, where absorption and reflection are present. Moreover, most peptides have no chromophores that absorb in the visible range—they show substantial absorption only in the UV range. However, absorption and reflection, even without spectroscopic analysis, provide the contrast mechanism for light microscopy (see above).

### 3.4. Emission Spectroscopy and Microscopy (Fluorescence and Raman)

The (theoretical) zero background makes emission techniques very attractive for imaging and spectroscopy.

Most peptides are not fluorescent (or only in the inconvenient UV); therefore, the method is mainly known for fluorescently labeled entities, such as nanoparticles and cells, which are used in post-modification. In this case, one can distinguish two basic setups. The standard optical microscope with fluorescence detection is a very fast imaging tool, while confocal laser excitation is a scanning method, working pixel by pixel, which means it takes seconds to minutes for a single image. Both provide means to localize fluorophores at high resolution, but well above the diffraction limit.

Raman spectroscopy involves excitation of the fiber with a strong laser and analysis of the inelastically scattered light. Although the setup is principally similar to confocal laser fluorescence microscopy, the Raman process has a much lower yield than fluorescence, and thus requires very sensitive detectors and filters (usually optical gratings). Specifically, scanning pixel by pixel is a slow process, and sufficiently resolved images can take hours of operation time.

The Raman process excites vibrations (comparable to IR) and hence analyzes functional groups in a sample [24,25,28]. Raman scattering can provide an even better vibrational characterization than IR; for example, at very low vibrational energies, phonons and complex molecular vibrations down to 100 cm^−1^ are easily accessible. Most important for electrospun fibers, laser excitation combined with a confocal setup translates into a very high resolution, easily reaching the diffraction limit. No specific sample preparation is required; thus, Raman microscopy should be the method of choice, if it were more widespread.

A very interesting extra feature is the polarization of the incoming beam, and polarization analysis of the scattered light. Depending on the addressed vibration, it can be possible to extract average orientations of functional groups with respect to the fiber axis, even for noncrystalline samples. Finally, ROA (Raman with circularly polarized light), never applied for electrospun peptides, distinguishes enantiomers, and is complementary to CD in solution.

A drawback of all Raman techniques is beam damage, which can be extreme for all organic matter. Raman spectroscopy should always be considered as a destructive technique. The proper choice of laser wavelength can help, but more often the laser intensity has to be reduced, resulting in very slow accumulation. The low thermal conductivity of peptides means that pulsed or chopped lasers might not be helpful concepts to reduce damage.

### 3.5. X-ray Diffraction

X-ray diffraction (XRD) is not frequently used for electrospun fibers because most of the usual polymer materials are not crystalline. This is also true for proteins—most proteins require elaborate procedures for crystallization. Electrospinning, due to the solvents, tends to unfold proteins [41], which does not allow for crystallization either. Peptides have to assemble during the electrospinning process, and given their small size, chances are much higher to form crystallites. There are very few relevant experiments with powder XRD (samples in random orientation), but these general ideas appear to be correct, as shown for poly(phenylalanine) [99] and for a synthetic alpha-helical protein (E. Georgilis et al., in preparation): The diffractograms of the peptide powder and of the electrospun fibers are nearly identical. It is suggested to test single stretches of fibers with microscale diffraction techniques, which are unfortunately not widespread. Beam damage, well known from synchrotron XRD, must be considered, however.

### 3.6. Atomic Force Microscopy

**Figure 14:**
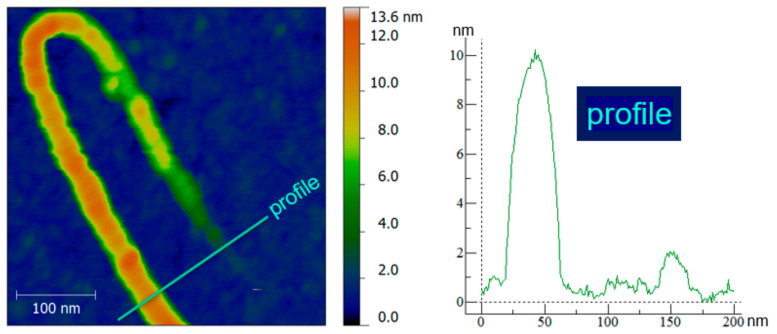
AFM analysis of ultrathin electrospun ovalbumin. Courtesy of Dr. A. Eleta Dr. W. Nuansing, CIC nanoGUNE.

While AFM is a staple in solid state analysis, scanning fibers is a challenge: AFM is tailored to flat surfaces, not to fibers with a (more or less) circular cross-section. Except for ultrathin fibers, AFM is better suitable to detect nanoscale surface features on the very top of fibers. Differently from SEM or Raman, AFM does not damage the samples, not even soft ones, as long as oscillatory modes (“noncontact”, “tapping”, etc.) are chosen. Then, AFM provides true 3D shape information of electrospun fibers (Figure 14) [27,41], with scanning times of minutes up to tens of minutes per image. Most electrospun fibers are rather smooth, thus AFM does not always add new information. Resolution on the molecular scale (1 nm) is very hard to achieve, even on ultraflat samples, therefore AFM is not the method of choice for the determination of molecular order and orientation.

### 3.7. Mechanical Testing

Mechanical tests are staples in materials science. AFM, positioned on a small region of interest, detects forces as a function of tip–fiber distance. In this way, nanomechanical testing and nanoindentation can be applied. They can yield various properties such as hardness, stiffness, and the related Young’s modulus. The latter is defined by the slope of stress (force per unit area) vs. strain (proportional deformation). To the best of our knowledge, relevant measurements have not yet been carried out, or they have been restricted to very specific cases [45]. On the millimeter sale, stress/strain mechanical tests are routinely employed to test samples of electrospun mats. The results, although unknown for peptides, determine the suitability for cell cultures or as biomaterials, for example. In both cases, high flexibility is required, and hence low elastic and bending moduli. This can be difficult to combine with high strain at failure, which is required when the fiber mat is used as a biomaterial in tissue repair [100].

## 4. Conclusions

In conclusion, starting from early successful examples of synthetic and natural electrospun polymers, (e.g., large proteins such as silk, chitin, and collagen) [12,13,101], attention in the last two decades has grown toward the use of peptides as the main or sole spun fiber component. The self-assembling ability of peptides is at the base of the potentiality of these low molecular weight compounds to be electrospun. With respect to polymers, peptides present several advantages, including reducing or eliminating animal-source materials, the possibility to be prepared at a large scale and, very importantly, to tune amino acid sequences for a desired purpose.

On the other hand, electrospinning of peptides requires very careful optimization of the electrospinning parameters, and it was observed that most fibers do not exhibit the desired mechanical properties. Sometimes they suffer from a lack of stability in biological environments and are not proteolytically stable, but the latter issue can be overcome by using non-natural amino acids in the sequence. Another important aspect, especially when moving into true applications, is the use of less toxic materials; specifically, the avoidance of fluorinated solvents.

In summary, the use of peptides to generate electrospun fibers is at an early stage of development. Further investigations and a thorough analysis of the forces involved in the assembly induced by the electric field are challenging issues for researchers aiming to find ways to improve the quality of spun fibers, mostly for biological applications. On the other hand, moving from polymers to peptides, important advances can be expected, when fibers are synthesized with new properties that diverge beyond biological applications, thus reaching out to fields such as energy storage or environmentally friendly materials.

## Figures and Tables

**Table 1:** Optimal conditions for electrospinning of peptides (TFA: trifluoroacetic acid) [25].

Peptide	Flow Rate (mL/h)	Concentration (wt.%)	Voltage (kV)
Phe-Tyr	0.05	18%, 80/20:TFA/HFIP	12 kV and 16 kV
Trp-Tyr	0.1	18%, 30/70:TFA/HFIP	12 kV and 16 kV
Tyr-Tyr	0.02	18%, 70/30:TFA/HFIP	12 kV and 16 kV

**Table 2:** Discussed polypeptide acronyms.

Acronym	Polypeptide
PGA	poly(l-glutamic acid)
PBG	poly(γ-benzyl-l-glutamate)
PLO	poly(L-ornithine)
PLEY	poly(L-glutamic acid_4_-co-L-tyrosine_1_)
γ-PGA	poly(γ-glutamic acid)
PBGA	poly(γ-benzyl-l-glutamate)-r-poly(l-glutamic acid)
PBGA20	copolymer containing 20 mol% poly(l-glutamic acid) and 80 mol% poly(γ-benzyl-l-glutamate)
PBGA30	copolymer containing 20 mol% poly(l-glutamic acid) and 80 mol% poly(γ-benzyl-l-glutamate)
PBGA20Na	Sodium salt of PBGA20

**Table 3:** Characteristics of electrospun fibers discussed.

	Sequence	Solvent	Electrospinning Conditions	Comments	Ref.
**Short Peptides**	Phe-Phe	HFIP	17.5 wt.% solution 13.2 kV 13.8 cm distance from collector	Hydrophobic, crystalline fibers of infinite length and sub-micron diameter	[24]
	Fmoc-Gly		>15 wt.% 10–30 kV 10–20 cm	Fibers of 100–300 nm in diameter	[23]
	Fmoc-Phe-Gly			Long amorphous fibers composed of smaller needles. Diameter of the needles: 300–400 nm	[23,41]
	Phe-Tyr	HFIP/TFA mixtures	18 wt.% 12 and 16 kV 10–20 cm	Fibers of 50–200 nm average diameter	[25]
	Trp-Tyr				
	Tyr-Tyr				
**Amphiphilic Peptides**	1: Me(CH_2_)_14_-Glu_2_-Val_3_	Water	3 wt.% 10 kV 5 cm	Fibers of 3.8 μm wide composed of nanoribbons	[59]
	2: Me(CH_2_)_14_-Val_3_-Ala_3_-Glu_3_			Fibers of 3.8 μm wide composed of cylindrical nanofibers	
**Peptidomimetics**	3: tBuCO_2_-(pyrazole-isothiazole core)-Gly-Fmoc	HFIP	30 wt.% 15 kV 15 cm	Continuous, fully filled fibers, 600 nm in diameter	[27]
**Polypeptides**	PLO	Water	20–60% *w*/*v* 5–20 kV 5–15 cm	Fibers 0.5–1.5 μm in diameter	[74]
	PLEY			Fibers 0.7–9 μm in diameter	[74]
	γ-PGA modified with Phe	HFIP	20 wt.% 20 kV 20 cm	Superhydrophobic Fibers 660 nm in diameter	[21]
	PGA	THF/DMAc mixture	20 wt.% 20 kV	Fibers 1–2 μm in diameter	[82]
	PBG				
	PBGA				
	PBGA20				
	PBGA20Na				
	PBGA30				
**Polypeptoids**	PPGly (with PEG)	Methanol/Water mixture	1.5 and 5 wt.%. of PEO (900 and 100 kDa, respectively) 10 wt.% PPGly 20 kV 25 cm	Fibers 680 nm in diameter, 520 nm after PEG annealing	[90]
